# Are routine tuberculosis programme data suitable to report on antiretroviral therapy use of HIV-infected tuberculosis patients?

**DOI:** 10.1186/1756-0500-6-23

**Published:** 2013-01-18

**Authors:** Miranda Brouwer, Paula Samo Gudo, Chalice Mage Simbe, Paula Perdigão, Frank van Leth

**Affiliations:** 1 Health Alliance International, Technical Assistance Unit, Maputo, Mozambique; 2 Ministry of Health, National TB Programme, C.P. 264 Av. Eduardo Mondlane/Salvador Allende, Maputo, Republica de Moçambique; 3 Ministry of Health, Provincial Health Directorate Manica Province, Chimoio, C.P. 264 Av. Eduardo Mondlane/Salvador Allende, Maputo, Republica de Moçambique; 4 Independent chest physician, Maputo, Mozambique; 5 Department of Global Health, Academic Medical Center, University of Amsterdam, Amsterdam Institute for Global Health and Development, Pietersbergweg 17, 1105 BM, Amsterdam, The Netherlands; 6 KNCV Tuberculosis Foundation, Postbus 146, 2501 CC, The Hague, The Netherlands

**Keywords:** Africa, Routine programme data, Tuberculosis, HIV

## Abstract

**Background:**

Antiretroviral therapy (ART) is lifesaving for HIV-infected tuberculosis (TB) patients. ART-use by these patients lag behind compared to HIV-testing and co-trimoxazole preventive therapy. TB programmes provide the data on ART-use by HIV-infected TB patients, however often the HIV services provide the ART. We evaluated whether the data on ART-use in the TB register were complete and correct. The timing of ART initiation was evaluated to assess whether reporting on ART-use could have happened with the TB case finding reporting. We collected data on TB treatment, HIV testing and ART for adult TB cases in 2007 from three TB clinics in Manica Province, Mozambique. These data on use of ART from TB registers were compared with those from the HIV services.

**Findings:**

Of 628 patients included, 504 (81%) were tested and of these 356 (71%) were HIV-infected. Of the co-infected patients, 81% registered with the HIV services in the same facility. The TB register was correct on ART-use in 73% of co-infected cases and complete in 74%. Information on ART-use could have been reported with the TB case finding reports in 56% of co-infected patients.

**Conclusion:**

The TB register is reasonably correct and complete on ART-use. However, the HIV patient record seems a much better source to provide this information. Reporting on ART-use at the end of the quarter in which TB treatment starts provides the programme with timely but incomplete information. A more complete but less timely picture is available after a year.

## Background

The use of the potentially life saving antiretroviral therapy (ART) for tuberculosis (TB) patients co-infected with the Human Immunodeficiency Virus (HIV) did not progress as much as would be necessary in addressing the dual TB and HIV epidemic. Globally, the HIV prevalence among new TB patients is 13%. In Mozambique this figure is 61% [1]. Worldwide, 29% of co-infected patients used ART in 2005, which had increased to 46% in 2010. In the same period, the percentage of TB patients knowing their HIV-status increased fourfold and provision of co-trimoxazole preventive therapy (CPT) reached 75% of HIV-infected TB patients.

The World Health Organization (WHO) reports these figures yearly in its global TB-control report. Although in many countries the HIV services provide ART, also to TB patients, national tuberculosis programmes (NTPs) provide the data for this report. However, it is not known how complete and correct the data on ART-use are within NTPs. This is relevant as access to ART is one of the main indicators of TB-HIV collaborative activities [2].

To use routine programme data to monitor progress on implementation, ideally the data are correct, complete and timely available [3]. WHO recommends reporting of HIV-testing for TB patients with the TB case finding data at the end of the quarter [4]. Data are thus available shortly after the end of the quarter. However, for CPT and ART-use the recommendation is to report with the treatment outcome data 12 months after the start of TB treatment. This is not timely reporting and precludes a timely response from the programme in case of under-utilization.

In Mozambique TB treatment staff offer HIV-testing to all TB patients and CPT to the co-infected since 2006. For further care and treatment including ART, they refer the co-infected patients to the HIV services. TB staff report both HIV testing of TB patients, and CPT and ART-use by the co-infected together with the TB case finding data at the end of the quarter. Therefore the data on CPT and ART-use are earlier available to the programme in comparison to the WHO recommended reporting timeline. This early availability can be useful for programme management purposes if the data are correct and complete.

This study therefore evaluated the correctness and completeness of the routine TB registers on ART-use among HIV-infected TB patients. We addressed the following questions: 1) How correct and complete are the data in the TB register on ART-use? 2) How complete could the data on ART-use be for reporting at the end of the case finding quarter?

## Methods

### Study design and setting

We selected purposefully three health facilities in Manica province, Mozambique. Criteria for selection included at least 150 TB patients notified in 2007 and the presence of both TB and HIV treatment services in the same facility. The participating health facilities were an urban facility in the provincial capital and two rural facilities about 20 and 80 kilometres from the provincial capital.

In the TB recording and reporting system of Mozambique all TB cases receive a unique number in the notifying facility’s TB register. Patients have their HIV test result and the start date of ART recorded in this register. All patients registered at the HIV clinic have a patient record, which contains clinical information, and a unique number for identification purposes. A link between the TB register and HIV patient record does not exist. The unique HIV patient number is not systematically recorded in the TB register, though TB staff is encouraged to do so. The three selected health facilities have, in addition to the government’s paper recording system, an electronic HIV patient database installed by an international non-governmental organisation.

### Data collection

We included all notified TB cases aged 15 years and older from January 1^st^ until December 31^st^, 2007 from the participating facilities. The facility’s TB supervisor collected the data using standardized forms. Firstly, data from the TB register were collected. These included: the start date of TB treatment, the HIV test result and initiation date of ART if the patient used ART.

Next, we used the unique HIV patient number, if available in the TB register, to identify the HIV records of the HIV-positive TB patients. In addition, local staff familiar with the patients identified some records. If these methods did not lead to identification of the HIV patient record, we performed a search in the electronic HIV-database using the patient’s name and age taken from the TB register. We took the unique HIV patient number from the electronic database and used it to locate the HIV patient record for positive matches. We limited the identification of the HIV patient record to those HIV-positive TB patients referred to the HIV services in the same health facility.

We collected CD4+ cell count results and dates from 12 weeks before and during TB treatment, ART-use, and the start date for ART from the HIV patient record.

To evaluate whether the TB register was correct and complete on ART-use, we compared the data on ART-use from the TB register with those of the HIV patient record. In case of discrepancy on ART-use, we took the data from the HIV patient record as correct for assessing ART-use because the HIV services provide ART. We also verified if the patient should have started ART according to the national guidelines for those co-infected patient not on ART. At the time of the study, co-infected patients were eligible for ART when their CD4+ count equalled or was below 350 cells/mm^3^[5]. We limited our analysis to the patients for whom both the TB register information and the HIV patient record were available.

We evaluated whether the patient started ART before or in the same quarter as TB was diagnosed to assess how complete the data could be at the end of the TB case finding quarter.

We used standard WHO treatment outcomes to define the end of TB treatment [6]. This was necessary to determine whether the patient initiated ART during the course of TB treatment.

### Statistical analysis

We used EpiData version 3.1 for data entry and performed descriptive analysis with EpiData Analysis V2.2.1.171.

### Ethics

The study protocol was approved by the National Bio-ethic Committee of the Ministry of Health of Mozambique and by the Institutional Review Board of the University of Washington in Seattle, USA. Because we used routinely available data, we did not obtain informed consent.

## Findings

**Figure 1:**
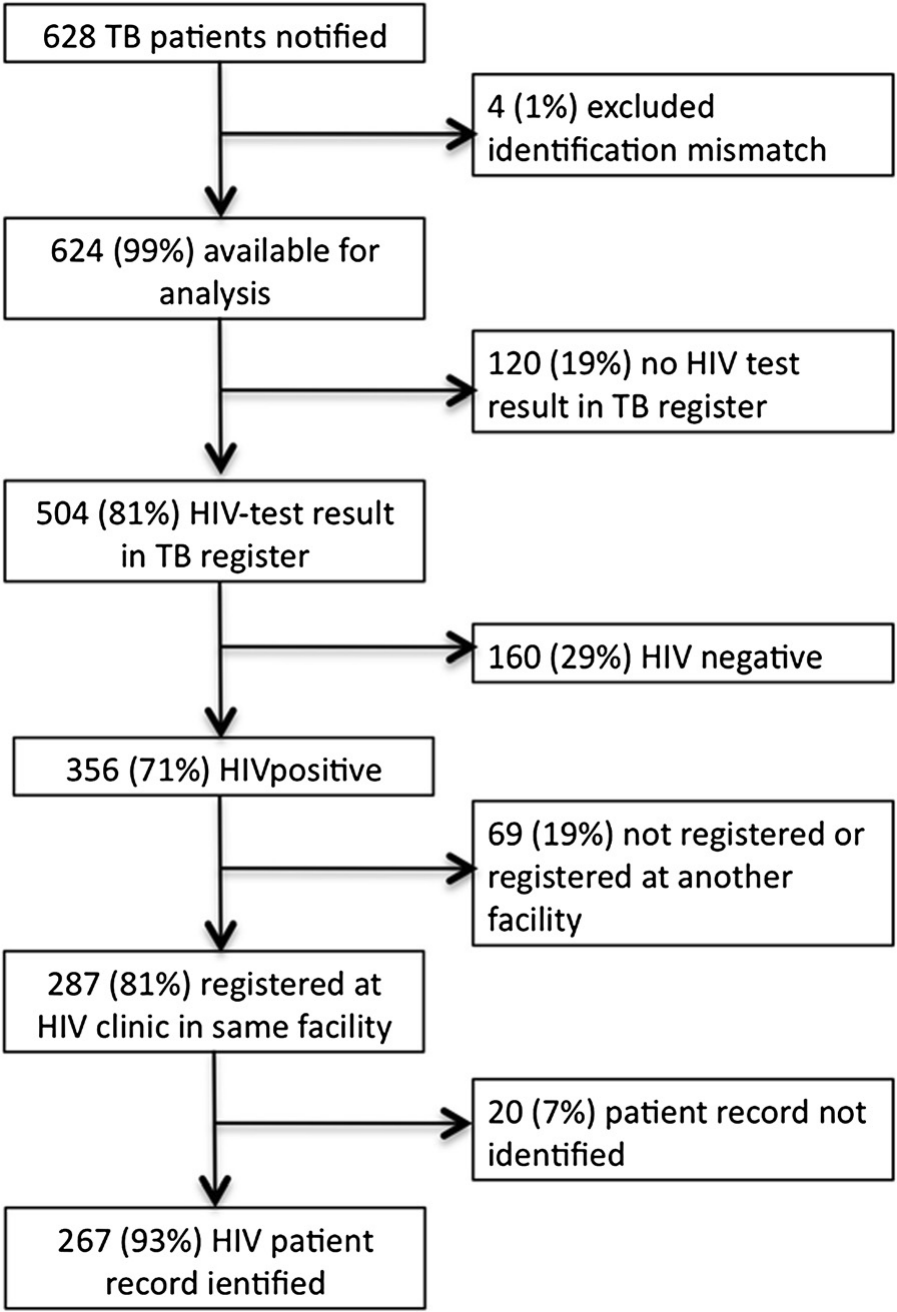
Breakdown of notified TB patients and their linkage to the HIV patient record.

Between January 1^st^ and December 31^st^, 2007, the three health facilities notified 628 TB patients of 15 years and older. Figure 1 shows a breakdown of the TB notifications for which we identified the HIV patient records (n = 267).Table 1 shows the TB-HIV data for the individual health facilities.

**Table 1:** Results of HIV testing, registration with HIV services and ART-use in the three health facilities

	**Facility A (Urban, in provincial capital, n = 153)**		**Facility B (Rural, 20 km from provincial capital, n = 245)**		**Facility C (Rural, 80 km from provincial capital, n = 226)**		**Total (n = 624)**	
	**Number**	**%**	**Number**	**%**	**Number**	**%**	**Number**	**%**
HIV-test result in TB register	147	96	170	69	187	83	504	81
HIV-positive (of those with HIV test result)	105	71	118	69	133	71	356	71
ART-use registered in TB register (of the HIV-infected)	16	15	59	50	86	65	161	45
Registration with HIV services in the same health facility (of the HIV-infected)	81	77	94	80	112	84	287	81
HIV Patient record available	74	91	83	88	110	98	267	93
ART-use in HIV patient record (of the HIV-infected)	47	45	59	50	70	53	176	49

Of 157 co-infected patients recorded in the TB-register as using ART, 130 (83) were confirmed when crosschecking the HIV patient record. Of the 110 co-infected patients recorded in the TB register not to be using ART, 64 (58%) were confirmed. In total, 194 (73%) entries of ART-use in the TB-register were correct.

The HIV patient records had 176 patients recorded as on ART. Of those, 130 patients had ART-use recorded in the TB register, a completeness of 74%.

In addition to the 176 patients on ART in the HIV patient record, 23 patients not on ART should have been on ART according to the national guidelines because they had CD4+ cell counts equal to or below 350 cells/mm^3^ during or before TB treatment. In total, 199 co-infected patients should have started or continued on ART whilst on TB treatment. Of these 199 patients, 150 (75%) could have been reported at the end of the quarter with the TB notifications. Potentially, the information in the TB register could have been complete for 150/267 (56%) at the end of the TB case finding quarter. When reported with the TB treatment outcomes, the TB register would have been complete on ART-use for 199 of the 267 (75%) co-infected patients.

## Discussion

The data on ART-use in the TB register were correct in 73% and complete in 74%. The reporting at the end of the case finding quarter could have been 56% complete, if data on ART-use from the HIV patient records were optimally transcribed in the TB registers.

The Global Fund to fight AIDS, Tuberculosis and Malaria assesses performance of grants on their programmatic achievements. The Global Fund considers a progress against target achievement between 60 and 89% as adequate [7]. Using this performance assessment, our study showed that the information of the TB register on ART-use had an adequate level.

However, there is much that can be improved in reporting on ART-use in co-infected patients. A reduction of the incorrect recording of patients in the TB register as using ART of whom ART-use was not confirmed by the HIV patient record would improve data quality. In our study we found this incorrect recording on ART-use in 17% of co-infected patients. We did not evaluate the reasons for this. Anecdotal evidence informed us that patients at times state they already use ART, whereas in fact they do not. Reasons why patients do not state their ART-use correctly might be even more complex than the reasons why patients do not start ART. A recent qualitative study in Malawi found that not starting ART was related to both health system and patient reasons [8]. Reasons in the former category included ART not being offered and non-availability of ART drugs. Fear of drug toxicity was the main patient related reason. The study showed that to implement the recommendation to start all co-infected patients on ART irrespective of the CD4+ cell count requires substantial efforts from the health system [9]. A study in Cameroon explained a test rate of 95% partly because patients usually follow health care providers’ recommendations [10]. This shows the importance of the health care provider offering ART.

In addition, more improvement could come from more complete recording of ART-use in the TB register. This study showed that 26% of co-infected patients were recorded in the TB register as not on ART but the HIV patient recorded showed they were in fact using ART.

The NTP in Mozambique reported an ART-use of 33% for the whole country in 2007 [11]. This was lower than the 73% correctly recorded co-infected patients in the TB register on ART that we found. We collected the data more than 12 months after TB treatment started, whereas the NTP data were reported at the end of the quarter in which TB treatment started. This partly explains the difference. Another reason could be that the HIV services provide ART and little collaboration or integration between the TB and HIV services existed. However, the health facilities have an ART committee of which the TB supervisor is a member. The committee discusses patients that are about to start ART and as such the TB supervisor potentially is aware of the ART status of the patient.

We found quite a difference between the individual health facilities on HIV testing and on ART-use. It is not clear where these differences come from. For HIV-testing, stock outs of HIV-test kits could be a problem. The urban health facility is close to the provincial distribution centre and maybe suffers less from stock outs or for a shorter period of time. The difference in ART-use seemed much more related to the data exchange between the two services. According to the TB register in facility A, only 15% used ART, while the HIV-records showed a use of 45%.

The reporting on ART-use at the end of the case finding quarter was estimated to be complete in only 56%. Reporting of ART-use with the TB treatment outcomes 12 months after the start of TB treatment increased completeness to 75%, but reduces the timely availability of information. Furthermore, in many countries the reporting on treatment outcomes is limited to sputum smear-positive cases whereas reporting on ART-use would need to be for all HIV-infected TB cases. This discrepancy may influence the quality of reporting on ART-use. Electronic data systems could facilitate data collection but also provide prompts to remind health care providers to take certain action, for example to collect a blood sample for a CD4+ cell count or to start ART [12]. In our study 12% of the co-infected patients did not have a single CD4+ cell count available during TB treatment. Potentially more patient would have been eligible to start ART.

Even though the data in the TB register on ART-use were adequately correct and complete, this study showed that services provided by one programme and reported data on these services by another programme lead to inconsistencies. Data reporting is not a goal on its own, but provides programmes with essential information on their performance. Ultimately, we want to improve patient outcome and integration of TB and HIV services may be the answer to that. A recently published systematic review showed that integration of ART provision into TB care seems to improve patients outcomes, however, the evidence is not yet sufficient [13].

## Limitations

This was a retrospective study based on routine data. Selection bias may have occurred by including only facilities where both TB and HIV services were present in one province in Mozambique. Potentially, ART-use may be higher in facilities with both services available than in facilities with only TB treatment and this may have led to a potential overestimation of the ART-use.

Another source of selection bias might be that not all HIV patient records could be traced. However, we have no evidence to assume that the presence of an HIV-record is associated with adequate reporting of data. Furthermore, we may have underestimated the completeness of data because we did not verify ART status of patients receiving ART in other health facilities than the one where they were treated for TB.

We considered the information on ART-use from the HIV patient record, partially from an electronic database, as correct. Potentially the HIV patient record contains incorrect data. However, a recent study from the same study area validated the electronic database used in our study [14]. That study found high levels of more than 95% agreement for the CD4+ cell count, the date of the CD4+ cell count and the start date of ART.

We collected the data 18 to 30 months after the start of TB treatment. The WHO recommended reporting on ART-use, is 12 months after the start of TB treatment. Our findings may present an overestimation of completeness though we think that little incentive exists for TB staff to update their registers after reporting the treatment outcomes.

## Conclusion

The data on ART-use in TB register are reasonably correct and complete. However, the TB register may not be the best source to report on ART-use during TB treatment because the information in the HIV patient record is much more accurate. Reporting on ART-use at the end of the quarter in which TB treatment starts provides the programme with timely but incomplete information. A more complete but less timely picture is available after a year.

Future research should focus on how to use optimally the available data in a way that does not add an extra burden to health care workers.

## Competing interests

The authors declare that they have no competing interests.

## Authors’ contributions

MB, PSG and PP conceived the study. All authors participated in the design of the study. MB and CMS coordinated data collection. MB and FvL participated in the statistical analysis. MB, PSG, PP and FvL drafted the manuscript. All authors read and approved the final manuscript.
